# Effects of neuromuscular training on postural control of children with intellectual disability and developmental coordination disorders: Neuromuscular training and postural control

**DOI:** 10.1186/s12891-022-05569-2

**Published:** 2022-07-02

**Authors:** Esmail Balayi, Parisa Sedaghati, Somayeh Ahmadabadi

**Affiliations:** 1 Adapted Physical Education, University of Guilan, Rasht, Iran; 2 Faculty of Physical Education and Sports Sciences, University of Guilan, Rasht, Iran; 3 Department of Sports Sciences, University of Farhangian, Tehran, Iran

**Keywords:** Dynamic balance, Functional balance, Hemsball training, Intellectual disability, Physioball training

## Abstract

**Background:**

Children with Intellectual disabilities who suffer from developmental coordination disorder represent insignificant physical fitness, strength, and balance. The prime objective of this research is to explore the impact of eight weeks of neuromuscular (combined physio-hemsball) training on postural control and balance of students with intellectual disabilities suffering from developmental coordination disorder.

**Methods:**

The present study was a prospective randomized clinical trial with a pretest–posttest design. The statistical population consisted of boys with intellectual disabilities, suffering from developmental coordination disorder randomly divided into two groups: the experimental group (*n* = 15) and the control group (*n* = 15). informed consent was obtained from all participants’ parents. Parents completed developmental coordination disorder questionnaires. Tests (Balance Error Scoring System, Y-Balance, timed Get Up & Go) were used to determine postural control, dynamic balance, and functional balance of subjects. The experimental group performed a combined physio-hemsball training for 8 weeks. Ethical considerations were observed according to the Helsinki Declaration and the CONSORT guidelines and regulations were followed to report this study.

**Results:**

Results showed that combined physio-hemsball training for 8 weeks can greatly improve postural control and dynamic and postural balance among students with intellectual disabilities suffering from developmental coordination disorder.

**Conclusions:**

According to the results, instructors can use this type of training to improve postural control and balance in boys with intellectual disabilities enduring developmental coordination disorders.

**Trial registration:**

This research was registered by the clinical trial centers of Iran (code IRCT20200125046254N1, Date of registration: 24/04/2020).

**Supplementary Information:**

The online version contains supplementary material available at 10.1186/s12891-022-05569-2.

## Introduction

According to the American Association on Intellectual and Developmental Disabilities (AAIDD), intellectual disability is a condition in which individuals experience below-average IQ [1]. The growing cycle 1 is calculated by investigators as 18 years old, thereby intellectual disability vulnerabilities that develop over 18 years of age are not taken into consideration [2]. Around three percent of the worldwide population becomes intellectually disabled referring to global statistical data [3]. Among those who are intellectually disabled, there are children who through their abilities to gain knowledge of reading and writing as well as rudimentary mathematics activities by instruction and experience, are distinct from one ‘s usual classmates. One of these discrepancies is regarded as developmental coordination disorder [4]. Generally, children who suffer from developmental coordination disorder are passive and inactive and also represent insignificant physical fitness, strength, stamina, balance, and coordination [5]. On the other hand, temporomandibular disorders which are common in children [6–9], especially TMJ disorders [10], should be noticed as important issues for children with intellectual disabilities.

Among these children's mobility impairments, weak posture controls are highly troubling, although keeping the balance of such children is more reliant on their sense of sight, triggering some sensory disruption, such as dropping, physical damage, or reduced activity or involvement by these children [11]. The preservation of strength, stamina, and dynamic balance in these individuals too is necessary to preserve a better quality of life as well as operational freedom [12]. Consequently, the implementation of physical exercise is one of the strategies for avoiding injury incurred by repeated slips and is mostly attributed to poor physical strength and imbalance of intellectual disability suffers [13] and nowadays is one of the new topics across the world of medical-sports investigation in the domain of core stability and hemsball. Core stability and hemsball have a variety of advantages, including improved sporting performance, injury prevention, strengthened limb coordination, and enhanced balance [14, 15]. While several experiments have demonstrated the impact of various interventions on the motor function of individuals with intellectual disabilities, limited researchers have focused on the effects of balance and modified training on the motor function of intellectual disability people with developmental coordination disorders. In two separate studies, Zolghadr et al. demonstrated the beneficial positive impact during eight weeks of selected balance-correction training on static and dynamic balance and motor strengthening function across individuals with intellectual disabilities with The developmental coordination disorder **(**DCD) [16, 17]. While other investigations have been undertaken either in healthy individuals enduring developmental coordination disorder or in mentally retarded individuals lacking developmental coordination disorder. In that respect, Dana et al., Afshari et al. acknowledged that core stability exercises strengthen balance and conserve the stature of intellectually disabled girls [18]. Also, in a survey, Najmehnejad et al. found a major impact of local indigenous games on the static and dynamic balance of learnable intellectually disabled students [19]. Stojanović et al. mentioned the positive effect of an exercise intervention program on the balance of intellectually disabled young individuals [20]. In an investigation, Christiansen et al. demonstrated a significant influence of selected exercises compared to strength exercises on postural control in children with developmental coordination disorders [21]. Shahrbanian and colleagues revealed the efficacy of core stability training on balance and reaction time of children experiencing developmental coordination disorder [22]. Balance and posture control are also extremely significant in the daily life of individuals experiencing intellectual disabilities. And the physical fitness of such patients relative to healthier people appears inadequate or requires attention as well as physical training; so the absence of physical fitness induces overweight, restricted motor function, decreased posture mobility and stability, and frequent falls [23]. Therefore, core stability manages to avoid the appearance of inappropriate movement patterns by maintaining appropriate balance and posture during functional activities, and on the other hand, hemsball training as an improving factor for bilateral balance and coordination, progress and strengthening upper limb coordination. Otherwise, it was also deemed as a fun and cheerful game to promote the impression of involvement of people in the competitiveness and cooperation of such children within communities and thereby to develop athletic success rates. Therefore, given that the balance will strengthen the sensory-motor and nervous-muscular system as well as the deep muscles that are connected to the core stability within the body, through these movement patterns exercises, consecutive tripping and fell can be prevented. According to these mentioned experiments, it is significant to address training sessions, which can concurrently influence the balance and posture control, as well as the coordination of these individuals. This research thus explores the effects of combined physio-hemsball training on postural control and intellectual disability of children suffering from developmental coordination disorders.

## Methods

### Participants

The study was experimental and applied with a pretest–posttest design with a control group. The population of this study consisted of boys with intellectual disabilities. After obtaining the school official permissions, the subjects' personal information and medical records were collected. informed consent was obtained from all participants’ parents. To determine the sample size, with dynamic balance(mean ± SD = 54.45 ± 2.11), G*POWER statistical software was used with test power (0.80), effect size (0.80), and significance level (0.05) [17]. There are four categories of intellectual disability which differ by IQ score- mild (IQ 50–55 to ~ 70), moderate (IQ 35–40 to 50–55), severe (IQ 20–25 to 35–40), and profound (IQ < 20–25) [24]. In this study, 52 boys categorized by the school’s healthcare assistant with mild intellectual disability (6—13 years), were eligible to enter the study.

Because students with mild intellectual disability who study in special schools do not show serious physical problems and can take care of themselves independently. Although they are able to learn daily living skills, they show delayed walking and talking skills in comparison with their peers [24].

Twenty two students were excluded from the sample based on the inclusion criteria and the results of the developmental coordination disorder list. This step of sample selection was one-way blind and non-randomized. Finally, 30 students were divided randomly into two groups: experimental (*n* = 15) and control (*n* = 15). The inclusion criteria consisted of intellectual disability with IQ of 55 to 70, intellectual disability with developmental coordination disorder, gender of subjects (boys), not using of neuroleptics or affecting on balance, not having a history of lower limb injuries and the surgery during the last one year, not having any disease in the arterial system and not cochlear implants, not having visual impairment and having normal vision without using glasses and the desire and ability to participate in the test as well as parental satisfaction. The exclusion criteria consisted of not having the developmental coordination disorder, no need for any support or the use of assistive devices to balance and walk, the development of musculoskeletal pain during the test, having ankle injuries, lower limb and spine surgery in the past year, a history of neuromuscular diseases, severe hearing and vision problems, and taking neuroleptics.

Before the pre-test session, the written consent and DCD questionnaire were completed by students’ parents. The pre-test evaluation of participants was conducted regarding the postural control, functional balance, and dynamic balance using the Balance Error Scoring System (BESS) test, Y-Balance test, and Timed Get Up & Go test, respectively. The experimental group then performed combined physio-hemsball training for 8 weeks, 3 weekly sessions, and one hour for each session. During the research, the control group in the school carried out only their daily activities. At the end of eight weeks, similarly to the pre-test phase, all parameters across both groups were once again calculated.

**Fig. 1:**
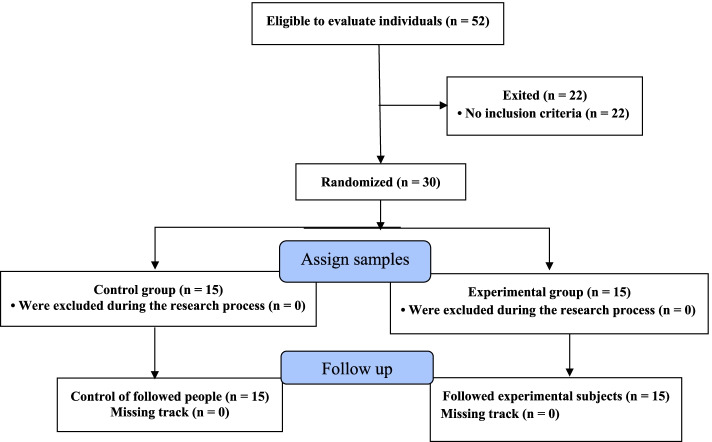
The CONSORT flowchart of the study and the process of the allocation of subjects to research groups

Figure 1 shows the CONSORT flowchart of the study and the process of the allocation of subjects to research groups. Also, reporting checklist for the randomised trial Based on the CONSORT guidelines used for this study, can be found in Additional file 1. All ethical considerations were observed according to the Helsinki Declaration.

### Measures

#### Evaluation of postural control

Balance Error Scoring System (BESS) test was exploited to evaluate static postural control with 75% validity and 50% to 82% reliability [25, 26]. The static balance test was accomplished in three standing positions including both feet parallel position, standing on one leg (dominant leg and non-dominant leg), and tandem stance (standing with one leg forward and one leg back). Besides, the dominant leg was determined to measure balance tests by the desire to kick a soccer ball [27]. The hands were over the pelvis in all three positions and the evaluation was carried out while the eyes closed. These three positions were managed to perform for 20 s for each position on two rigid surfaces (ground) and a compliant surface (foam). When implementing the experiment in each position, six varieties of human errors were counted and documented for each assignment. These mistakes involved removing the hands from the waist, attempting to open the eyes, stepping or falling, trying to lift the heel or toe, bending the torso forward or sideways more than 30 degrees, and shifting away from the defined position for 5 s of each measurement. Each experiment was repeated three times [2, 28].

#### Dynamic balance assessment

To measure the dynamic balance, a Y-balance test with 91% validity and 84% reliability was exploited [29]. In this test, 3 orientations (anterior, posterior-external, posterior-internal) were distinguished with an angle of 135 degrees from each other. As respects this experiment has a significant relationship with the length of the foot, to fulfill it and normalize the practical information of the foot, it was measured from the upper anterior iliac spine to the inner ankle in a supine position lying on the ground [29]. Each participant practiced the experiment six times to gain knowledge on how to execute the test. The participant stood on one foot in the center of the seat and with the other foot in the direction selected by the investigator, carried out the full output procedure without failure, and returned to the original correct position. To mitigate the effect of an intervention, each participant exercised each trajectory 6 times, including 15 s for rest at a time. After a five-minute rest, the subject began the test in the direction randomly selected by the examiner, and the examiner measured the contact point of the person's foot to the center of the thumb in centimeters. The test was repeated three times for each subject and the best record was divided by the length of the leg and then multiplied by 100 to obtain the achievement distance in terms of the percentage of leg length. After a five-minute break interval, the respondent started the examination in the position selected randomly by the investigator, and the instructor calculated the contact point of the individual's foot to the center of the thumb in centimeters. In the occurrence of a foot mistake in the center, the experiment was replicated. The subject conducted three tries in each orientation and the sum of these current efforts was taken into consideration to be a dynamic balance ranking [30].

#### Performance balance evaluation

Timed Get Up & Go test (TUG) was applied. To conduct this assessment, several chairs without arms, a stopwatch, and a trajectory of three meters are required. The three-meter distance started from the basis of the chair. The participant sat on a chair and leaned on the back of the chair while wearing his usual clothing and shoes. With the command of the test taker, the participant gets up and walks the marked three meters. After hitting the finish point, he turned around and sat back on a chair (he travels a count of Six meters). The period of the experiment was documented in seconds as an individual's performance, so it is important to keep in mind that the rating for this experiment is an average of three tests that were completed, and 91% validity and 99% reliability were recorded [31]. Individuals whose test period in this assessment was less than 20 s have satisfactory independence movement [32].

According to investigations on balance training and postural stability and coordination in individuals with intellectual disabilities and developmental coordination disorder, the examined procedure demonstrated a high standard in terms of comprehensive movements and the utilization of mechanisms participating in postural balance and stability and coordination. Accordingly, the combined physio-hemsball training procedure [15, 33], was exerted as the training intervention. The control group did not encompass any training program during the survey duration, but the experimental groups accomplished the training principles for eight weeks, three sessions per week, and 1 h per session. Firstly, the participants warmed up for 10 min (7 min of walking and 3 min of stretching) and then were segregated into two groups, and with the command of the trainer, group one began the training program and after the end of each movement, group two began the identical training program and same movements. Eventually, cooling down was carried out for 5 min (2 min of walking and 3 min of stretching). All training was performed inside the school gym. The purpose of the game is to throw the ball into the ring (like such a circular pattern) without touching the hoop as well as preventing it from entering the region of the opponent. While throwing the ball into the game and the opponent's zone, they may catch the ball in any manner, including the plates on which they stand. The serving player launches the ball into the hoop and delivers it towards the opponent. This counting lasts till the ball hits the inside of the hoop, drops out or the player commits an error. The service scorer is the player who earns a point. The player who wins a point makes the service. The play zone is 1.23 × 3 cm for young players and 1.55 × 4 cm for adults, and for two-player players, 2.10 × 4.15 cm the playground is rectangular, flat, smooth, and rigid. This region should be regulated so that it is 2 m away from the ball throwing zone in each direction. When throwing the ball, the entire body rotates across the trajectory of the objective, and the eyes are concentrated on the objective point, and the hand that throws the ball to the hoop moves forward while the foot is lifted in the identical direction of the ground and the shoulders brought ahead. When the ball gets out of hand, the arm keeps driving the ball in the trajectory of the ball. These training were routinely implemented and were characterized orally by the investigator previous to the training and are detailed within Table 1.

**Table 1:** Combined physio-hemsball training protocol

	First and second week (3 × 10)	Third, fourth, fifth week (3 × 12)	Sixth, seventh and eighth week (3 × 15)
**Warm up**	10 min: Mid-paced walk 7 min and Stretching 3 min		
	**Basic coordination exercises**	**Wall coordination exercises**	**Two-person coordination exercises**
**Coordination training**	**Exc.1:** Roll the ball from one hand to the other through the palm of the hand **Exc.2:** Throw the ball from one hand to the other with palms facing each other and held at a hand’s distance **Exc.3:** Release the ball from the fingertips while pushing the arm upwards **Exc.4:** Release the ball to the ground while standing, catch it with one or both hands **Exc.5:** Catch the ball which has been released to the ground while standing with one or both hands while hunkering **Exc.6:** Dribble the ball during every second step while walking	**Exc.1:** Catch a ball that is thrown against a wall and bounces back while in a sitting position facing the wall with one or both hands (1–2-3 m) **Exc.2:** Hold the ball while standing up facing the wall and catching it with the same or a different hand **Exc.3:** Catch a ball that is thrown onto the ground and bounces back from the wall with one or both hands while standing and facing the wall **Exc.4:** Catch a ball with one or both hands that is thrown into a hoop left in front of a wall and rebounds from the wall	**Exc.1:** Two students with intellectual disabilities standing in alignment facing the wall catch a ball thrown onto the ground and rebound from the wall with both hands **Exc.2:** An intellectual disability student catches a ball that is thrown directly at the wall by the trainer and falls to the ground from the wall **Exc.3:** The trainer standing behind the intellectual disability student facing the wall throws the ball on the ground and the ball rebounding from the wall is caught by the intellectual disability student **Exc.4:** An intellectual disability student standing on a foot plate catches a ball that is thrown by the trainer at different speeds **Exc.5:** Two intellectual disability students face each other on the foot plates in the game area and throw a ball at the target board with one hand and catch the ball with two hands **Exc.6:** The trainer and student practice throwing on the game court on one foot and on two feet
**Physiobal training**	1-Upper-body roll out 2-Inclined press-up 3- Contralateral single-leg hold 4- Quadruped exercise	1-Upper-body roll out 2-Inclined press-up 3- Contralateral single-leg hold 4- Quadruped exercise	1-Upper-body roll out 2-Inclined press-up 3- Contralateral single-leg hold 4- Quadruped exercise
	3 set with 3 repetitions Hold time 4 s 30 s rest	2 set with 3 repetitions Hold time 4 s 30 s rest	1 set with 3 repetitions Hold time 4 s 30 s rest
**Cool down**	5 min: Low-paced walk 2 and min Stretching 3 min		

To measure the normality of the information, the Shapiro–Wilk test was exploited, and to analyze the pre-test and post-test outcomes, a dependent t-test and covariance analysis were exerted at a significance level of *p* < 0.05 and the pretest was used as a covariate factor from the covariance test in SPSS software version 25.

## Results

Table 2 presents the demographic characteristics of participants in both groups. According to the results, both groups were homogenous in terms of demographic characteristics. The subjects were normalized in terms of age, height, weight and BMI in the target groups. The Shapiro–Wilk test evaluated data normality and based on this test’s results, variables of overall score of postural control, dynamic balance, and functional balance were normal (*p* > 0.05) and two variables of Stand on a hard surface, Stand on the foam surface were not normal (*p* ≤ 0.05). Therefore, normal and non-normal variables were tested by parametric (Analysis of covariance) and nonparamentric tests (Mann–Whitney U) respectively.

**Table 2:** Demographic characteristics of the study samples

P	T	Mean ± SD	Group	**Measurement index**
0/15	1/44	9/20 ± 1/24	Control	**Age (y)**
		10/06 ± 1/83	Intervention	
0/08	1/79	1/37 ± 0/10	Control	**Height (m)**
		1/44 ± 0/12	Intervention	
0/2	1/31	36/95 ± 10/47	Control	**Weight (kg)**
		42/26 ± 11/65	Intervention	
0/55	0/59	19/24 ± 2/34	Control	**BMI (kg/m2)**

To evaluate the average outcomes between the two groups, considering the pre-test as a factor covariance, the covariance experiment was employed, which represented a remarkable variation in the maximum rating of postural control, dynamic balance, and functional balance (*P* ≤ 0.01) (Table 3). Due to the abnormality of the BESS test score, the non-parametric Wilcoxon-Mann–Whitney test was exerted to investigate the variations between the control and experimental groups, which demonstrated a considerable difference between the two groups in the BESS test (*P* < 0.01) (Table 4). To survey the variations between pre-test and post-test in the two groups separately, the paired sample t-test (parametric variables) was handled which presented a substantial difference in the overall rating of postural control, dynamic balance, functional balance (*P* < 0.01) (Table 5). To investigate the shifts in pre-test and post-test in the two groups separately, the Wilcoxon signed-rank test (non-parametric variable) was applied. There was a remarkable difference between the two groups (*p* < 0.01) but there was no substantial discrepancy in parallel feet standing on both rigid and compliant surfaces (*p* ≥ 0.01) (Table 6).

**Table 3:** Comparison of post-test variables between group

**Variable**	**Measurements**	**group**	Mean*	**F**	**df**	**p-value #**	**Eta Squared**
**Overall score of postural control**	Post-test	Control	6/42	36/9	1	0/0001^**^	0/57
	Post-test	Experimental	3/39				
**Dynamic balance**	Post-test	Control	48/04	12/56	1	0/0001^**^	0/31
	Post-test	Experimental	63/28				
**Functional balance**	Post-test	Control	7/4	14/45	1	0/0001^**^	0/34
	Post-test	Experimental	6/63				

^#^ Analysis of covariance * Pre-test (covariate agent) ** Significance at the level of *P* < 0.01

**Table 4:** Comparison of postural control variables in pre-test and post-test between groups

	**Variable**	**Stage**	**z**	**U**	***p*-value #**
**Stand on a hard surface**	Standing on two legs next to each other	Pre-test	-0/58	102/00	0/68
		Post-test	-1/79	90/00	0/36
	Standing on one foot	Pre-test	-1/22	87/00	0/30
		Post-test	-4/41	9/50	0/001
	Standing tandem	Pre-test	-0/24	107/00	0/83
		Post-test	-3/41	32/5	0/001
**Stand on the foam surface**	Standing on two legs next to each other	Pre-test	-1/08	90/00	0/36
		Post-test	-2/39	75/00	0/12
	Standing on one foot	Pre-test	-1/73	80/00	0/18
		Post-test	-4/38	10/5	0/001
	Standing tandem	Pre-test	-0/24	107/5	0/83
		Post-test	-3/21	38/00	0/001

^#^ Mann–Whitney U

**Table 5:** The difference between the mean of the variables in the before and after training sessions

group Variable	Control Group (15 n)				Experimental Group (15 n)			
	Pre-test	Post-test	T	p-value	Pre-test	Post-test	T	*p*-value
**Overall score of postural control**	5/8 ± 1/79	6/35 ± 1/57	-1/21	0/24	6/38 ± 1/32	3/45 ± 1/12	6/21	0/001^*^
**Dynamic balance**	50/76 ± 13/52	46/21 ± 14/00	1/14	0/27	56/14 ± 12/13	65/12 ± 14/6	-4/82	0/001^*^
**Functional balance**	7/77 ± 0/94	7/73 ± 1/17	-1/38	0/22	7/02 ± 0/94	6/3 ± 0/71	6/97	0/001^*^

**Table 6:** (mean difference) of different posture control modes in subjects before and after training session

	**group** **Variable**	**Control group (15 n)**				**Experimental group (15 n)**			
**position**		Pre-test	Post-test	Z	*p*-value	Pre-test	Post-test	Z	*p*-value
**Stand on a hard surface**	Stand on a hard surface	1/00 ± 1/92	1/00 ± 2/17	00/0	1/00	0/46 ± 0/99	0/001 ± 0/001	-1/63	0/10
	Standing on one foot	9/00 ± 1/81	9/00 ± 1/81	00/0	1/00	7/86 ± 2/66	4/13 ± 2/16	-3/43	0/001^*^
	Standing tandem	7/86 ± 3/02	8/06 ± 2/76	-0/63	0/52	7/53 ± 3/46	3/86 ± 1/99	-3/32	0/001^*^
**Stand on the foam surface**	Stand on a hard surface	1/8 ± 2/9	1/6 ± 2/87	-0/7	0/48	0/8 ± 1/52	0/001 ± 0/001	-1/84	0/06
	Standing on one foot	9/66 ± 1/04	9/53 ± 1/3	-1/41	0/15	8/53 ± 2/16	6/00 ± 2/13	-3/45	0/001^*^
	Standing tandem	8/46 ± 2/47	8/93 ± 1/98	-1/34	0/18	8/86 ± 1/88	6/66 ± 1/79	-3/57	0/001^*^

## Discussion

The objective of this research was to examine the impact of eight weeks of combined physio-hemsball training on postural control and balance of students suffering from developmental coordination disorders. The research results revealed that eight weeks of combined physio-hemsball training had a substantial influence on the total rating of postural control, dynamic balance, functional balance and across various postural control modes only in one-foot standing positions and tandem standing in both rigid and compliant surfaces, but the effect of parallel feet standing on both rigid and compliant was not noticeably variant. The findings from this existing survey in terms of dynamic balance and functional balance alongside the outcomes of several experiments have indicated that physical training and core stability improve and progress the static and dynamic balance of mentally disabled children [16, 19, 25, 34, 35] which is consistent with the above research. But in terms of dynamic balance, it contradicts the investigation of Hosseini et al. [36] and in terms of functional balance, it is also in contrast with the research of Zolghadr et al. [16]. The origin of the discrepancy is attributable to discrepancies in the balance measurement, length, strength, and form of the training procedure. However, the involvement of physical activity or preferably sports can contribute to enhancing motor function among individuals experiencing developmental coordination disorder, however, according to a review of related literature, the impact of combined physio-hemsball training on dynamic balance and functional balance as well as postural control was not observed among individuals experiencing intellectual disability. Since one of the features of children with developmental coordination disorder is manifested in physical training variables, including coordination and motor balance, developing motor skills is challenging for these individuals [37]. Concerning these difficulties and, based on study findings, combined physio-hemsball training illustrated positive influences on balance. In a review survey, the positive effects of a training intervention on improving and strengthening the balance of youth individuals experiencing intellectual disabilities were noticed, which was in constant with the present investigation. Therefore, based on the existing findings of this research, it can be concluded that training as a non-pharmacological method can effectively enhance the balance-related difficulties regarding individuals with intellectual disabilities suffering from developmental coordination disorder. Besides, through the social involvement that occurs across sporting and sports environments, physical training can improve vitality and self-confidence as well as decrease the isolation of this group of people experiencing disabilities. However, decreased social participation can increase the rate of mental and emotional disorders among people with intellectual disabilities and consequently worsen physical problems. Therefore, social and physical disability have been the causes of further physical and emotional illness [9, 38].

Another result obtained is significance in the postural control variable. In an investigation by Christensen et al. (2018), they mentioned the beneficial impact of selected activities in comparison to strength training on postural control among children with developmental coordination disorder, which is consistent with the existing investigation. As a result, there is a rationale for improving core stability and postural balance as well as postural control [21]. Although the vestibular system is the most critical sense for postural control and is commonly considered a prerequisite for the development and improvement of motor skills. A deficiency in the balance of intellectual disability people is presumably to delay and hinder their motor skills and also their motor function development [39].

Among the essential parameters requiring receiving information from visual, atrial, and sensory-somatic nervous systems and vestibular inputs. Consequently, this information must be processed and integrated based on the individual's circumstances and the situation in which they are positioned to preserve the balance [15]. Researchers investigating the balance in these individuals have revealed that people with intellectual disabilities have more fluctuations and variability in maintaining balance than normal individuals, demonstrating a weaker and insufficient balance in this group [40].

In the current investigation, it can be argued that implementing combined physio-hemsball training has culminated in better and more reliable interpretation and accurate processing of input information from the mentioned systems. Considering the continuity of training for eight weeks, a more appropriate and acceptable integration between input data from various resources has been carried out. The outcome has shown the stronger conservation and stability of static and dynamic balance among these children. Because in the combined physio-hemsball training of the current survey, in order to accomplish the movements accurately, it was intransitive to constantly coordinate visual feedback and deep sense. On the other hand, the cerebellum performs an indispensable role in forecasting activities and adjusting motions to motor functions. Another excellence of combined physio-hemsball training is a modern enjoyable and engaging activity that can be experienced and performed through individuals of any age and also can be learned and played at household with close relatives. It is highly essential to have good hand–eye coordination, deep focus to grab the ball and throw it while playing Hemsball [15]. It appears, however, that the routine practice of these movement patterns has strengthened the balancing function of individuals. As an outcome, all motor function assessments of individuals experiencing intellectual disabilities can be efficient and coordination/balance and avoidance of repetitive falls can be improved. Owing to the significance of static and dynamic balance in the daily life of individuals suffering from intellectual disabilities and the acceptance of the principle that balance is a quite significant reliable indicator for the daily life activities of individuals with mental disabilities, due to the cost-effectiveness and accessibility of these equipment types of training, these drills can be carried out conveniently at home by the person. availability of these exercises [41].

### Limitations

The present study has some limitations which should be considered. Firstly, the limited sample size was the main limitation because the number of eligible boys based on the inclusion criteria- intellectual disability with developmental coordination disorder- was limited in the research area. Secondly, very little relevant research has been done regarding intellectual disabilities in children. Therefore, associated studies are required to provide this group of children with useful motor function exercises.

## Conclusions

The results demonstrated that eight weeks of combined physio-hemsball training could have remarkable effects on postural control, balance and bilateral coordination, and upper limb coordination of the intellectual disability individuals beating developmental coordination disorder. Therefore, instructors and trainers can employ these training patterns in their rehabilitation and sports activities, during sports hours, and in schools to progress the balance and postural control of these children for developing their motor functions, and also improve their self-confidence. This combined physio-hemsball training is also effective in increasing their independence and provides them a basis for welfare and participation across society.

## Supplementary Information

**Additional file 1. **CONSORT 2010 checklist of information to include when reporting a randomised trial

## Data Availability

All data generated or analyzed during this study are included in this published article.
